# Molecular basis of vitamin K-dependent protein γ-glutamyl carboxylation

**DOI:** 10.1038/s41422-025-01185-6

**Published:** 2025-09-29

**Authors:** Qihang Zhong, Dandan Chen, Jinkun Xu, Yao Li, Wanqiong Yuan, Yan Meng, Qi Wen, Qiwei Ye, Guopeng Wang, Kexin Pan, Chunli Song, Lin Tao, Jie Qiao, Jing Hang

**Affiliations:** 1 State Key Laboratory of Female Fertility Promotion, Center for Reproductive Medicine, Department of Obstetrics and Gynecology, Peking University Third Hospital, Beijing, China; 2 National Clinical Research Center for Obstetrics and Gynecology (Peking University Third Hospital), Key Laboratory of Assisted Reproduction, Ministry of Education (Peking University), Beijing key Laboratory of Reproductive Endocrinology and Assisted Reproduction, Beijing, China; 3 Department of Biophysics, School of Basic Medical Sciences, Peking University, Beijing, China; 4 School of Basic Medical Sciences, Peking University, Beijing, China; 5 Beijing Academy of Artificial Intelligence, Beijing, China; 6 School of Life Sciences, Tsinghua University, Beijing, China; 7 Department of Orthopedics, Engineering Research Center of Bone and Joint Precision Medicine, Ministry of Education, Beijing Key Laboratory of Spinal Disease Research, Peking University Third Hospital, Beijing, China; 8 State Key Laboratory of Membrane Biology, School of Life Sciences, Peking University, Beijing, China; 9 Department of Orthopedics, First Hospital of China Medical University, Shenyang, Liaoning China; 10 Institute of Advanced Clinical Medicine, Peking University, Beijing, China

**Keywords:** Cryoelectron microscopy, Post-translational modifications

Dear Editor,

Gamma-glutamyl carboxylase (GGCX) is the sole endoplasmic reticulum (ER) membrane-embedded human enzyme that catalyzes γ-carboxylation of glutamate (Glu) to γ-carboxyglutamate (Gla), coupled with oxidation of vitamin K hydroquinone (VKH_2_) to its 2,3-epoxide form (VKO).^1–3^ This modification of vitamin K-dependent proteins (VKDPs) is essential not only for coagulation but also for vascular calcification, bone metabolism, tumor proliferation, and spermatogenesis.^4,5^ All VKDPs share a conserved N-terminal propeptide for GGCX binding^6^ and a downstream GLA domain harboring Glu.^7^ GGCX deficiencies lead to multiple clinical phenotypes exemplified by vitamin K-dependent coagulation factor deficiency;^8–10^ however, the molecular mechanisms governing its enzymatic activity and the associated pathologies remain incompletely understood. Here we present high-resolution cryo-EM structures of human GGCX bound to vitamin K and coagulation factor IX (FIX) or factor X (FX). By integrating biochemical and cellular assays with molecular dynamics (MD) simulations, we elucidate the molecular basis of VKDP exosite binding and the dual-catalytic properties of GGCX. These findings offer a comprehensive molecular framework for understanding vitamin K metabolism in physiology and disease, and provide valuable insights into potential therapeutical intervention.

Co-expression of wild-type (WT) GGCX (GGCX^WT^) with full-length FIX in HEK293F cells and the following cryo-EM analysis yielded a 2.78 Å GGCX^WT^•FIX complex structure (Fig. 1a; Supplementary information, Fig. S1a–f and Table S1). The overall architecture of GGCX comprises an N-terminal transmembrane domain (TMD, TM1–TM9), a luminal propeptide-binding domain (PBD) with two α-helices (H1 and H2), and a flexible Arch-like domain containing H3–H6 (Fig. 1b, c; Supplementary information, Fig. S2a). Helix H3 aligns parallel to the membrane, H4 is oriented perpendicular, and H5 runs along the detergent micelle surface (Fig. 1b). The cryo-EM density clearly resolved both the FIX propeptide and GLA domain (residues 29–92) within the luminal domain cavity, along with a distinct density corresponding to endogenous vitamin K surrounded by TM5–TM8 and the elongated loop connecting TM5–TM6, surrounding lipids and glycosylation sites (Fig. 1a–c; Supplementary information, Fig. S2b–d). In vitro γ-carboxylation assays with a truncated FIX variant (FIXQ/S) confirmed GGCX enzymatic activity only in the presence of VKH_2_, as further validated by concentration- and time-dependent assays (Fig. 1d, e; Supplementary information, Fig. S3a–d). Surface plasmon resonance (SPR) analysis demonstrated high-affinity binding between GGCX and FIXQ/S, with a dissociation constant (*K*_d_) of 252 nM (Supplementary information, Fig. S3e).

**Fig. 1:**
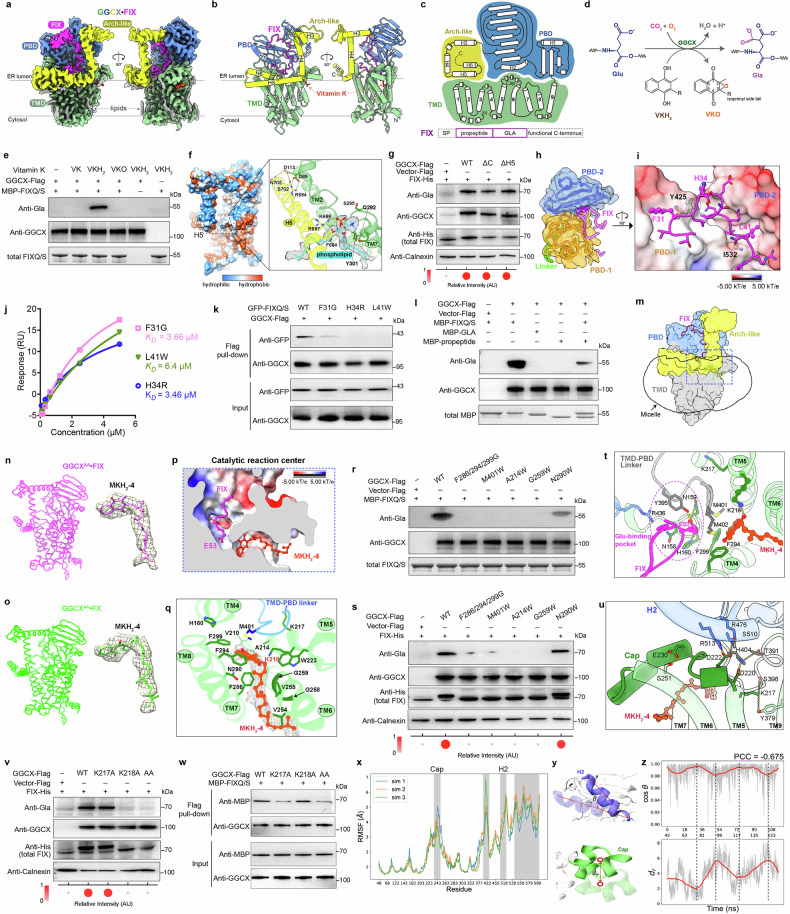
Structural and mechanistic investigation of human GGCX. **a** Cryo-EM density of GGCX bound to FIX and vitamin K in a membrane-embedded orientation. Individual domains are labeled and color-coded. **b** Cartoon representation of GGCX^WT^•FIX complex in the same orientation as in **a**. **c** Topology of GGCX (TM1–9 and luminal helices H1–H6) and schematic diagram of FIX including signal peptide (SP) and GLA region. **d** Scheme of GGCX-catalyzed γ-carboxylation, converting Glu to Gla, coupled with oxidation of VKH_2_ to VKO. **e** In vitro enzymatic assay of GGCX in the presence of different vitamin K forms. MBP-fused FIXQ/S was used. Representative images from at least three biological repeats are shown. **f** Hydrophobic surface of GGCX, highlighting H5 interactions with lipids and adjacent TM2. A phosphatidylcholine is shown. Hydrogen-bonds and salt bridges are annotated as yellow and cyan dashed lines, respectively. **g** Cell-based γ-carboxylation assays of GGCX truncation constructs. ΔC, C-terminal deletion (residues 1–728); ΔH5, H5 deletion (residues 1–676). Calnexin served as an internal ER control. Empty vector (vector-Flag) served as negative control. The carboxylation ratio (carbonylated to total GGCX) was semi-quantified as the intensity of the anti-His upper band divided by the sum of the upper and lower bands. **h**, **i** FIX-propeptide is clamped inside the groove formed by PBD-1 and PBD-2 (**h**), with the close-up view displayed with PBD as electrostatic surface (**i**). **j** SPR analysis of binding affinities (*K*_d_) between GGCX and mutated FIXQ/S. **k** Pull-down assays with Flag-immobilized GGCX and GFP-fused FIXQ/S mutants. **l** In vitro competition carboxylation assay using MBP-fused FIX. **m** Positioning of FIX within the ER luminal cavity with the reaction center highlighted. **n**, **o** Cryo-EM structure of GGCX-K217A/K218A double mutant (GGCX^AA^) in complex with FIX (GGCX^AA^•FIX) (**n**) or FX (GGCX^AA^•FX) (**o**). Densities and fitted models of MKH_2_-4 are displayed. **p** Cutaway view of the catalytic reaction center. **q** Detailed interactions for MKH_2_-4 with its density map shown as mesh. **r**, **s** In vitro (**r**) and cell-based (**s**) γ-carboxylation assays of vitamin K binding mutants of GGCX. F286/294/299G, triple mutation to glycine. **t** Location of the Glu-binding pocket adjacent to the vitamin K molecule. **u** Interactions between the Cap (D220–S246) on TMD and the H2 on PBD-2. **v** Cell-based assays of GGCX constructs. AA, K217A/K218A double mutant. **w** Pull-down assays with Flag-immobilized GGCX and MBP-fused FIXQ/S. **x** RMSF plot of GGCXΔArch from three independent aMD simulations. Cap and H2 are highlighted. A few residues from the N-terminus or loop regions were excluded. **y** Quantification of the motion angle of H2 (cos*θ*) and the distance for Cap movement (*dr*). Initial reference positions are rendered as transparent overlays. **z** Temporal evolution of cos*θ* and *dr* during aMD simulations. The original data shown as grey transparent lines and the smoothed trajectories as red lines. Two metrics are aligned after compensating for temporal delay with peaks and valleys marked by black dashed lines.

The PBD is organized into two lobes, PBD-1 and PBD-2, connected by a flexible linker that engages extensively with the TMD (Supplementary information, Fig. S4a). This interface is stabilized by multiple interactions, including a disulfide bond (C450–C99), hydrogen bonds (D442/Q446–K154), and salt bridges involving R476 whose mutation is linked to disease^11^ (Supplementary information, Fig. S4b, c). Several other key residues involved in hydrogen-bonding within the PBD, such as W501, Q503, G558, and T591, are also associated with pathological variants (Supplementary information, Fig. S4d). Mapping known GGCX mutations onto the structure disclosed two major hotspots: one at the catalytic center and the other at the PBD-1/PBD-2 interface (Supplementary information, Fig. S4e and Table S2), suggesting that stabilization of PBD is indispensable for GGCX function.

Arch-like domain exhibits inherent flexibility and contains an amphipathic H5 that is anchored parallel to the membrane surface and stabilized through both lipids and neighboring TMD residues (Fig. 1f). At one end, a high-resolution density tentatively assigned as phosphatidylcholine is anchored with its polar headgroup sandwiched by F684/R687/K688 on H5 and Q292/S295/Y301 on TM7 (Fig. 1f, right panel; Supplementary information, Fig. S2d). A well-resolved co-purified cholesterol was also observed (Supplementary information, Fig. S2d). Its persistence after multiple purification steps suggests a potential role in GGCX stability.^12^ At the opposite end, H5 is anchored by salt bridge between R694 and D89 and hydrogen-bonds between S702/N705 and D113 (Fig. 1f, right panel). The intrinsic flexibility of the Arch-like domain was further investigated by MD simulations using a GGCX^WT^–FIXQ/S–vitamin K system. The simulations revealed a rapid inward collapse of H3 and H4 (Supplementary information, Fig. S4f and Video S1). Quantitative analysis of per-residue root-mean-square-fluctuation (RMSF) indicated minimal dynamic fluctuations (~1 Å) across most regions of the protein, whereas the Arch-like helices and the C-terminus displayed substantial deviations (RMSF > 5 Å), resulting in an overall root-mean-square-deviation (RMSD) of ~7 Å (Supplementary information, Fig. S4g, h). These findings highlight the conformational plasticity of the GGCX luminal domain, particularly in the Arch-like region. We then performed cell-based γ-carboxylation assays using GGCX truncation mutants. C-terminal deletion (ΔC) or truncation up to H5 (ΔH5) caused only modest activity reduction; meanwhile, the γ-carboxylation of FIX induces a slight increase in its molecular weight, manifested as a subtle upward shift in the band mobility of total FIX (Fig. 1g; Supplementary information, Fig. S5a). To assess the function of Arch-like domain, we compared carboxylation activity with various substrates. ΔH5 showed no effect on FIX and FX, whereas deletion of the C-terminus or H5 markedly impaired FVII, GAS6, and protein S carboxylation (Supplementary information, Fig. S5b). These findings suggest that the conserved H5 is not only crucial for the luminal assembly but may also regulate γ-carboxylation in a substrate-dependent manner.

Next, we examined the interaction between propeptide and PBD. PBD-1 and PBD-2 form a claw-like groove that clamps FIX (Fig. 1h), functioning as an exosite^4^ spatially distinct from the catalytic center (Fig. 1b). Within this interface, multiple hydrophilic interactions occur: H34^FIX^ forms a π–π stacking with Y425^GGCX^, the benzene ring of F31^FIX^ inserts into a hydrophobic pocket in PBD-1, and L41^FIX^ establishes hydrophobic contacts with PBD-2 (Fig. 1i). Sequence alignment showed strong conservation at F31 but variability at H34 among VKDPs, yet their carboxylation activities were comparable (Supplementary information, Fig. S6a–c). To investigate their functional relevance, we performed site-directed mutagenesis. F31G, H34R, and L41W mutants exhibited markedly reduced binding affinities to GGCX (*K*_d_ of ~3–6 μM), > 10-fold weaker than WT (Fig. 1j; Supplementary information, Figs. S3e, S6d). Pull-down assays utilizing Flag-immobilized GGCX and GFP-tagged mutant FIXQ/S showed a corroborative result that all mutations disrupted propeptide binding (Fig. 1k). In vitro and cell-based assays with mutant GGCX showed that Y425A impaired carboxylation activity, while the pathologic I532T mutant exhibited a milder yet detectable reduction in activity (Supplementary information, Fig. S6e, f). A competition carboxylation assay indicated that neither MBP-fused GLA domain (MBP-GLA) nor propeptide alone (MBP-propeptide) underwent carboxylation; moreover, excess free MBP-propeptide competitively inhibited FIXQ/S carboxylation (Fig. 1l), indicating that propeptide facilitates substrate binding by competing for the same interaction site on GGCX. Collectively, these results demonstrate that the propeptide enhances GGCX-mediated carboxylation by stabilizing substrate binding.

The GLA domain is accommodated within an internal cavity formed by the luminal domain and TMD (Fig. 1m). A distinct density embedded within an elongated TMD pocket locates in close proximity to E53 of FIX, which we identified as vitamin K. Given the high abundance of menaquinone-4 (MK-4) in kidney and the use of HEK293F cells, we presumed the co-purified molecule to be predominantly MK-4, which was further validated by liquid chromatography-mass spectrometry (LC-MS/MS) (Supplementary information, Fig. S1b). To verify this assignment, we generated a catalytically inactive GGCX double mutant (K217A/K218A, GGCX^AA^), considering the established role of K218 as the active base for deprotonating VKH_2_.^13^ Structures of GGCX^AA^ in complex with FIX (GGCX^AA^•FIX) and FX (GGCX^AA^•FX) were determined at 2.59 Å and 2.58 Å, respectively (Supplementary information, Fig. S7 and Table S1). Both maps provided well-defined EM density for unambiguous assignment of the bound molecule as MK-4 hydroquinone (MKH_2_-4) (Fig. 1n, o). Superimposing GGCX^WT^•FIX onto GGCX^AA^•FIX facilitated modeling of MKH_2_-4 into the WT binding pocket.

A cutaway surface view illustrates that the vitamin K and GLA pockets are interconnected, cooperatively constituting the catalytic reaction center (Fig. 1p). MKH_2_-4 orients parallel to the membrane, with its 2-methyl-1,4-naphthoquinone head deeply buried inside a hydrophobic tunnel and its isoprenyl tail extending outward (Fig. 1m, p). The naphthoquinone ring is coordinated by a hydrophobic pocket involving F286^TM7^, F294^TM8^, F299^TM8^, H160^TM4^, V210^TM5^, M401^TMD–PBD linker^, and W223^TM5^ (Fig. 1q). The isoprenyl tail traverses a narrow (~11 Å) tunnel lined by A214^TM5^, G258^TM6^, G259^TM6^, V254^TM6^, V255^TM6^, and N290^TM7^ (Fig. 1q). To validate this site, we engineered mutations disrupting the hydrophobic pocket (F286/F294/F299G), introducing steric hindrance near naphthoquinone ring (M401W), or obstructing the isoprenyl tunnel (A214W, G259W, and N290W). Most mutations severely impaired the γ-carboxylation activity in vitro and in cells, except N290W, which retained partial activity (Fig. 1r, s). Furthermore, these mutations reduced vitamin K binding affinity by ~10-fold relative to the WT (*K*_d_ of ~0.85 μM) (Supplementary information, Fig. S8). Binding analysis of different forms of vitamin K showed stronger binding affinity for hydroquinone (vitamin K menaquinone (VK)) (*K*_d_ of ~1.02 μM) than its epoxide form (VKO) (*K*_d_ of ~8.19 μM) (Supplementary information, Fig. S9). These findings provide compelling evidence that these residues are essential for creating the vitamin K-binding pocket, and indicate that the weaker binding of VKO to GGCX facilitates the release and turnover of oxidated VK.

Regarding GLA binding, the γ-carboxyl group of E53^FIX^ inserts into a positively charged pocket adjacent to K218 and the naphthoquinone ring of MKH_2_-4 (Fig. 1t). K218 forms a hydrogen bond with the naphthoquinone ring; concurrently, N159, H160, and Y395 coordinate the E53 side chain, and N158 and R436 stabilize the backbone of GLA domain, creating the Glu-binding pocket. H160A mutation has been shown to compromise Glu carboxylation,^14^ underscoring its role in stabilizing transition state. The nearby F294^TM4^, F299^TM4^, M401^TMD–PBD linker^, and M402^TMD–PBD linker^ contribute to a “hole-in-the-wall” that physically separates the Glu- and vitamin K-binding pockets (Fig. 1t). Although K217 was proposed to stabilize the carbanion group of K218,^4^ our structure reveals that its ε-amino group is coordinated by hydrogen bonds between S398^TMD–PBD linker^ and Y379^TM9^, positioning ~12 Å away from the Glu carbanion (Fig. 1t, u). Additionally, TMD–PBD linker is embedded within the membrane, making direct interactions between K218 and Glu unlikely (Fig. 1t, u). These observations suggest that the primary function of K217 is stabilizing the interaction between the TMD and PBD-2. Results from activity assays, pull-down, and SPR binding analyses on two mutants indicated that while K218A abolished the catalysis of FIX without affecting substrate binding, K217A impaired VKDP binding without disrupting catalysis (Fig. 1v, w; Supplementary information, Fig. S10). These results demonstrate that K218 is critical for the catalytic reaction, whereas K217 primarily mediates conformational coupling independent of K218, highlighting their distinct and complementary roles in regulating GGCX activity.

Surrounding the vitamin K-binding pocket, the TM5–TM6 loop forms a Cap shielding it from the ER luminal solvent (Fig. 1u). The interaction of this Cap with H2^PBD-2^ is stabilized by an extensive hydrogen-bond network involving R513–D222–R476–S510, D220–H404–T391, E230–S251, and Y379–K217–S398 (Fig. 1u). We hypothesized that Cap motion is coupled with H2 movement during vitamin K binding release. To explore this hypothesis, we performed MD simulations using a GGCXΔArch–FIX–MK-4 system. Accelerated MD (aMD) simulations revealed remarkable conformational transitions in H2 and Cap, whereas the rest of the structure remains rigid (RMSF < 1 Å), in contrast to the minimal changes in conventional MD (Fig. 1x; Supplementary information, Fig. S11a, b and Video S2). To quantify these movements, we proposed two metrics: the cosine of the angle between H2 and its initial orientation (cos*θ*), and the deviation of Cap’s center of mass from its initial position (*dr*). A 160-ns aMD simulation trajectory exhibited the intermittent H2 opening and the Cap conformational transitions (Fig. 1y). Notably, their movements were not synchronized, exhibiting a temporal delay of ~45 ns. After removing this delay, a strong correlation emerged between the two motions with a Pearson correlation coefficient of –0.675 (Fig. 1z; Supplementary information, Fig. S11c). Cryo-EM structure comparison further showed that while the TMD remained unchanged, PBD-2 in the GGCX^AA^ complexes adopted an ~5° outward rotation relative to GGCX^WT^•FIX (Supplementary information, Fig. S11d), likely from disrupted coupling due to K217A mutation. This suggests that H2 acts as a conduction element coupling Cap conformational transition and PBD-2 rotation, establishing a specific H2-Cap transduction mechanism. Although attempts to resolve apo GGCX were unsuccessful, probably due to significant conformational flexibility (Supplementary information, Fig. S12a, b), we superimposed our GGCX^WT^•FIX structure with two reported apo structures.^12,15^ Without VKDPs, PBD-2 rotates ~20° away from the active center, disrupting the H2–Cap interface and creating a more open conformation for substrate binding (Supplementary information, Fig. S12c). Upon propeptide engagement, PBD-2 rotates inward to form hydrogen bonds with Cap, and subsequent vitamin K binding renders the ternary complex catalytically competent. Structures of GGCX bound to BGP, FIX, and FX are highly conserved, with K217 and K218 remaining essentially unchanged (Supplementary information, Fig. S12d). These findings further support the critical role of K217 in mediating VKDP binding and of K218 in catalysis.

In conclusion, this study delineates the molecular basis and the coupling mechanism underlying Glu γ-carboxylation and vitamin K epoxidation by GGCX (Supplementary information, Fig. S12e). Our structural and functional analyses not only shed light on the catalytic mechanism but also elucidate the functions of key pathological mutations, highlighting potential applications in advancing clinical anticoagulant therapy and broader aspects of cellular regulation.

## Supplementary information

Supplementary information

Supplementary information, Video S1

Supplementary information, Video S2

## Data Availability

The cryo-EM maps of GGCX•FIX, GGCX^AA^•FIX, and GGCX^AA^•FX have been deposited to EMDB with accession codes of EMD-62862, EMD-62863, and EMD-62864, respectively. The corresponding atomic models have been deposited to PDB with accession codes of 9L6Q, 9L6R and 9L6S, respectively.
