# Supplementary material for: Molecular basis of vitamin K-dependent protein γ-glutamyl carboxylation
Source: Cell Res. 2025 Sep 29;35(11):917–20. doi: 10.1038/s41422-025-01185-6 (PMC12589487; doi:10.1038/s41422-025-01185-6)
Supplement: Supplementary file 1 — Supplementary information [file 41422_2025_1185_MOESM1_ESM.pdf]

Supplementary information, Figures and Tables

Molecular basis of vitamin K-dependent protein γ-glutamyl carboxylation

Qihang Zhong[1,2#], Dandan Chen[1,3#], Jinkun Xu[4#], Yao Li[5,6#], Wanqiong Yuan[7#], Yan Meng[7], Qi Wen[1,2], Qiwei Ye[5], Guopeng Wang[9], Kexin Pan[8], Chunli Song[7]✉, Lin Tao[8]✉, Jie Qiao[1,2]✉, Jing Hang[1,2,10]✉

Materials and Methods

Transient protein expression in mammalian cells

The codon-optimized cDNAs encoding the full-length human gamma-glutamyl carboxylase (GGCX) and coagulation factor IX (FIX) or factor X (FX) were subcloned into the pCDNA3.1(+) vector, incorporating a C-terminal Flag tag for GGCX and a C-terminal His_6 tag for FIX or FX. Site-directed mutagenesis was performed via a standard two-step polymerase chain reaction (PCR) in combination with homologous recombination. Proteins (either GGCX alone or in complex with FIX or FX) were transiently expressed in HEK293F cells (Invitrogen). In brief, cells were shaken in SMM 293T-II medium (Sino Biological Inc.) at 37°C with 5% CO_2 in a Multitron-Pro incubator (Infors). When the cell density reached 2.0×10^6 cells/mL, transfection was initiated by adding 2 mg of total plasmid DNA (1:1 mass ratio for co-expression) and 4 mg of polyethylenimine hydrochloride (MW 40,000; Cat# 24765-1, Polysciences) per liter of culture. After 48 hours, cells were harvested by centrifugation at 2000 rpm for 20 minutes and stored at -80 °C for subsequent purification.

Recombinant protein purification

For structural and enzymatic studies, the thawed HEK293F cell pellets were thoroughly resuspended in lysis-A buffer (20 mM HEPES-K pH 7.4, 150 mM NaCl, 1 mM $MgCl_2$, and 1 mM EDTA) supplemented with 10% Glycerol. The suspension was further supplemented with 1% (w/v) Glyco-diosgenin (GDN; Cat# GDN101, Anatrace), protease inhibitor cocktail (Cat# 4693132001, Roche), and DNase (Cat# R017414,

RHAWN), and mixed homogeneously. The mixture was incubated at 4℃ for approximately two hours, followed by centrifugation at 30,000 rpm for one hour. The clarified supernatant was applied to ANTI-FLAG® M2 Affinity Gel beads (Cat# A2220, Sigma) pre-equilibrated with the lysis-A buffer containing 0.01% GDN, and incubated for an hour at 4℃. After washing three times with lysis-A buffer supplemented with 0.01% GDN, 0.1mM PMSF, and 1 mM ATP, bound proteins were eluted with lysis-A buffer containing 0.2 mg/mL Flag peptide. Elutes were concentrated to approximately 2 mL using a 100kDa cutoff ultrafiltration tube (Millipore), followed by size-exclusion chromatography using a Superose-6 Increase column (10/300 GL, Cytiva) equilibrated in lysis-A buffer with 0.01% GDN. The peak fractions were concentrated, flash-frozen in liquid nitrogen, and stored at -80°C for further study.

Substrate protein purification in *E.coli*

Codon-optimized cDNAs for the vitamin-K dependent proteins (VKDPs) were cloned into the bacterial expression vector pET-21c(+), with each construct engineered to encode an N-terminal maltose-binding protein (MBP) tag followed His-tag. Recombinant protein expressions were induced in *E.coli* BL21(DE3) cells with 0.25 mM isopropyl β-D-1-thiogalactopyranoside (IPTG). Cells were harvested and resuspended in lysis-B buffer (20 mM HEPES-K pH 7.4, 150 mM NaCl, and 1 mM PMSF) supplemented with protease inhibitor cocktail. After sonication and centrifugation, the clarified lysate was incubated with pre-equilibrated Ni Smart Beads 6FF (Cat# SA036100, Smart-Lifesciences) for 15 minutes. Beads were washed with lysis-B buffer containing 20 mM imidazole, and bound proteins were eluted with lysis-

B buffer containing 250 mM imidazole. The eluted proteins were concentrated, followed by ion-exchange chromatography (SOURCE-15Q) with a 50 mM–1 M NaCl gradient in 20 mM Tris-Na, pH 8.0. Fractions containing the target proteins were collected, concentrated using a 30kDa cutoff ultrafiltration tube (Cat# UFC903096, Millipore), and subjected to gel filtration using a Superdex-200 10/300 GL column (Cytiva) in lysis-B buffer. The peak fractions were concentrated, flash-frozen in liquid nitrogen, and stored at -80°C in small aliquots.

Cryo-EM sample preparation and data acquisition

Holy-carbon gold grids (Quantifoil Au 400 mesh, R1.2/1.3) were glow-discharged using either a Solarus 950 plasma cleaner (Gatan) or a PELCO easiGlow (Ted Pella) prior to cryo-EM sample preparation. Freshly prepared $GGCX^{WT}$•FIX, $GGCX^{AA}$•FIX, and $GGCX^{AA}$•FX complexes (4 µL aliquots at 4 mg/mL) were applied to the glow-discharged grids, blotted with Whatman No.1 filter paper at a blot force of –2 and a blot time of 0.5 s at 4°C and 100% humidity, and plunge-frozen in liquid ethane using a Vitrobot Mark IV (Thermo Fisher Scientific).

For the $GGCX^{WT}$•FIX complex, the cryo-grids were screened on a 200 kV Talos Arctica equipped with a Ceta detector (FEI). Data collection was carried out using Titan Krios G3 (FEI) operating at 300 kV, equipped with a K3 Summit (Gatan). Images were acquired in super-resolution mode at a nominal magnification of 81,000×, with a calibrated pixel size of 1.07 Å and a dose rate of 15 e^{-}/s/pixel. A GIF BioQuantum energy filter (Gatan) with a 20 eV slit width was used. The defocus range was set from

–0.8 to –1.2 μm. The total exposure time was 4.58 s, with intermediate frames recorded every 0.14 s, yielding a total of 32 frames per micrograph.

For the GGCX^{AA}•FIX and GGCX^{AA}•FX complexes, grids were initially screened using a 200 kV Glacios^{TM} 2 microscope (Thermo Fisher Scientific) equipped with a Falcon 4i detector (Thermo Fisher Scientific). Data were collected on a 300 kV Krios G4 (Thermo Fisher Scientific) equipped with a BioQuantum detector. Images were recorded in super-resolution mode at a nominal magnification of 105,000×, with a calibrated pixel size of 0.85 Å and a dose rate of 17e^{-}/s/pixel. The slit width of BioContinuum GIF electron optics was 20 eV. The defocus range was set from –0.8 to –1.8 μm. The total exposure time was 2.00 s, and intermediate frames were recorded every 0.06 s. A total of 32 frames per image were acquired. All movies were recorded semi-automatically using EPU software. Data collection statistics are summarized in Supplementary information, Table S1.

Imaging processing

A total of 4,598, 13,529, and 6,911 movie stacks were recorded for GGCX^{WT}•FIX, GGCX^{AA}•FIX, and GGCX^{AA}•FX complexes, respectively. The cryo-EM data were processed using CryoSPARC.[1] Motion correction of the micrographs and the contrast transfer function (CTF) parameters were performed using patch motion correction and patch CTF estimation, respectively. Blob-picking, 2D classification, Ab-initio model reconstruction, heterogeneous refinement, homogeneous refinement, non-uniform refinement, and 3D classification were sequentially performed. Global CTF Refinement and Local CTF Refinement were also performed for further map

optimization. Final resolutions of the map were estimated using gold-standard Fourier shell correlation (FSC) at a correlation cutoff value of 0.143. Local Resolution Estimation yielded global map resolutions of 2.78, 2.59, and 2.58 Å for $GGCX^{WT}$•FIX, $GGCX^{AA}$•FIX, and $GGCX^{AA}$•FX complexes, respectively. Workflow of the data processes are illustrated in the Supplementary information, Figs. S1 and S7.

Model building and refinement

The initial model of GGCX generated by AlphaFold2[2] was fitted into the cryo-EM map using ChimeraX.[3] Residues for FIX and FX were manually added into the initial models and adjusted using Coot.[4] Ligand-restraints for vitamin K (menaquinone-4 or its hydroquinone form) for refinement were generated by phenix.elbow.[5] Model refinements of all three datasets against the corresponding maps were performed in PHENIX using real-space refinement with secondary structure and geometry restraints.[6] Bound lipid molecules (phosphatidylcholine or cholesterol) were also modeled. The final structures were validated through examination of the Clash scores, Molprobity scores and statistics of the Ramachandran plots by PHENIX.[7]

***In vitro* γ-carboxylation assay**

The *in vitro* γ-carboxylation activity of GGCX was assessed via immunoblot detection using truncated constructs comprising the propeptide and GLA region of substrates (i.e,, FIXQ/S for FIX, residues 29–92) that fused to maltose-binding protein (MBP) tag at its N-terminus. Menaquinone-4 (MK-4) (Cat# 47774, Supelco) was reduced to hydroquinone form (VKH_2) by 0.2 M dithiothreitol (DTT) via overnight incubation in 25 mM Tris-HCl, pH 8.0 and 0.5 M NaCl. Briefly, 500 nM (2.5ug) of apo

GGCX with Flag-tag at its C-terminus (GGCX-Flag) was incubated with 20 μM substrates (MBP-FIXQ/S) on ice for an hour. Subsequently, 250 μM of VKH_2 and 920 μM of $NaHCO_3$ were added, and the reaction mixture was adjusted to a total volume of 50 μL with lysis-A buffer. The reaction was carried out at 20°C for an additional hour, and 5μL aliquots of the reaction mixtures were resolved by 10% SDS-PAGE. γ-carboxylation of substrates was visualized by western blotting with an anti-Gla monoclonal antibody (1:200, Cat# 3570, BioMedica Diagnostics). Coomassie blue-stained total FIXQ/S and immunodetected GGCX using anti-GGCX antibody (1:1000, Cat#16209-1-AP, Proteintech) were used as internal loading controls. Reactions without GGCX-Flag enzyme served as negative control.

Cell-based carboxylation activity assay

HEK293T cells were cultured in high-glucose DMEM (Cat# C11995500BT, Gibco) supplemented with 10% fetal bovine serum (FBS; Cat# FBS-AU500, Newzerum) and 1% penicillin-streptomycin (Cat# 25200056, Gibco) at 37°C in a humidified incubator with 5% CO_2. For transient transfection, cells were seeded in 12-well culture plates (Cat# 3513, Corning) at 70% confluency. Cells were co-transfected with Flag-tagged GGCX, His_6-tagged full-length FIX (FIX-His) or other VKPDs, and VKOR at 2:2:1 mass ratio using Lipofectamine 3000 (Cat# L3000015, Thermo Fisher) according to the manufacturer's instructions. After 24 hours, 10 μM vitamin K1 (Cat# SV8160, Solarbio) was added to the medium. Following an additional 48 hours, cells were rinsed with ice-cold PBS (pH 7.4) and lysed in cold lysis-A buffer supplemented with 1% (w/v) GDN and protease inhibitor cocktail for one hour. The supernatant was collected after

centrifugation, and the total protein concentrations were quantified using the BCA assay (Cat# 23225, Pierce). The proteins were resolved by SDS-PAGE and subjected to western blotting. γ-carboxylated levels of FIX, expression levels of GGCX, total FIX, and calnexin were detected using anti-Gla, anti-GGCX, anti-His (1:2000, Cat# CW0286M, CWBIO), and anti-calnexin (1:1000, Cat# 2679S, Cell Signaling Technology) antibodies, respectively.

Pull-down assay

Pull-down assays were performed using Flag-tagged GGCX immobilized on FLAG beads and substrate FIXQ/S region fused to either GFP or MBP. For analyses of the FIX mutants (WT, F31G, H34R, and L41W) in Fig. 1k, mutated FIXQ/S were fused with super-folded GFP at its N-terminus and purified as described above. FLAG beads were captured with 2.5 μg of Flag-tagged GGCX by incubation at 4°C for 30 minutes. Subsequently, an excess amount of GFP-FIXQ/S was introduced and the mixture was incubated for an additional 30 minutes. After extensive washing, bound proteins were eluted with 0.2mg/mL FLAG peptide. The elutes were subjected to immunoblot detection using the anti-GGCX antibody and anti-GFP antibody (1:2000, Cat# SLAB3001, Smart-Lifesciences). For pull-down assays of GGCX variants (WT, K217A, K218A, K217A & K218A) in Fig. 1w, all the procedures remained identical, with the exception that MBP-FIXQ/S was employed and analyzed via western blotting using anti-MBP antibody (1:2000, Cat# E022240, EARTHOX).

Competition assay

The γ-carboxylation competition assay shown in Fig. 1l was conducted similarly to the *in vitro* carboxylation assay. VKDP substrates, including MBP-FIXQ/S, MBP-GLA (MBP fused to only the GLA region), and MBP-propeptide (MBP fused to only the propeptide region), were purified as described above. For the single-substrate reactions, 500 nM GGCX was pre-incubated on ice for an hour with 20 μM MBP-FIXQ/S, MBP-GLA, or MBP-propeptide. For competition reaction, an additional 20 μM of propeptide was included in the pre-incubation mixture before reaction. Carboxylation reactions were initiated by the addition of 250 μM VKH_2 and 920 μM $NaHCO_3$, and the subsequent procedures were identical to those described for *in vitro* assay.

Western blotting

Samples from *in vitro*, cell-based carboxylation reactions, and pull-down assays were resolved by SDS-PAGE and transferred to Polyvinylidene fluoride (PVDF) membranes (Cat# IPVH00010, Millipore). Membranes were blocked in 5% (w/v) skim milk (Cat# 232100, BD) at room temperature for one hour, and then incubated overnight at 4°C with the primary antibodies as indicated above. After three washes with TBST buffer (25 mM Tris, pH 8.0, 150 mM NaCl, and 0.05% (w/v) Tween-20), membranes were incubated for an hour with Horseradish Peroxidase (HRP) conjugated-secondary antibodies (1:10000, Cat# P03S02M or P03S01M, Gene-Protein Link). Immunoblotting was visualized using an automated chemiluminescence imaging system (Tanon).

Surface plasmon resonance

Surface Plasmon Resonance (SPR) experiments were conducted using a Biacore 8K+ instrument (Cytiva) to determine the equilibrium dissociation constants (K_D) for GGCX interactions with FIX and vitamin K. Recombinant GGCX was diluted to a final concentration of 10 ng/μL in sodium acetate solution (pH 5.5) with 0.01% (w/v) GDN. A total volume of 100 μL GGCX was immobilized on CM5 sensor chips via amine coupling.

For GGCX-FIX (WT, F31G, L41W, and H34R) interactions, FIXQ/S analytes (78 nM to 5 μM) were injected in single-cycle mode at a rate of 30 μL/min for 120 s, followed by 240 s of dissociation in running buffer (20 mM HEPES-K pH 7.4, 150 mM NaCl, 1 mM $MgCl_2$, and 1 mM EDTA) at the same flow rate. For GGCX-vitamin K interaction, DMSO-dissolved vitamin K (VK, 0.78 μM–400 μM) or vitamin K epoxide (VKO) were injected at a rate of 30 μL/min for 60 s in single-cycle mode, followed by the injection of the running buffer with 5% DMSO at a flow rate of 30 μL/min for another 60 s. All experiments were conducted at 25 °C and data were analyzed using the Biacore 8K+ Evaluation Software.

Liquid chromatography-mass spectrometry (LC-MS/MS) Analysis

The $GGCX^{AA}$•FIX was extracted using 300 μL of hexane/isopropanol (3:2). The top 180 μL of the organic phase was collected and dried, and the residue was redissolved in 100 μL methanol. Following centrifugation at 20,000 g for 10 min, 30μL of the supernatant was injected into an LCMS-8050 (Shimadzu) for LC-MS/MS analysis. Tandem separation was performed on Phenyl-Hexyl and C18 column with a mobile phase consisting of A: acetonitrile and B: methanol using the following gradient

program: 95% B (0-4 min) at 1.0 mL/min, 0% B (4-5.5 min) at 2.0 mL/min, 95% B (5.5-7 min), and re-equilibration to 95% B (6.5-7 min) at 1.0 mL/min. Mass detection employed atmospheric Pressure Chemical Ionization (APCI) in positive mode with the following parameters: Nebulizing Gas Flow 3.5 L/min, Interface Temperature 300℃, DL Temperature 250℃, Heat Block Temperature 300℃, Drying Gas Flow 0, Interface Voltage 5 kV, CID gas 250 kPa, Q1 Resolution low. Quantification was performed in multiple reaction monitoring (MRM) mode, with optimized transitions for vitamin K (m/z 445.2 [Parent ion] → 187.2 [Product ion]; collision energy -24 V), and analyzed against a calibration curve. ER-embedded protein Calnexin was purified using the same protocol as GGCX and served as a negative control.

Simulation system design and configuration

Two molecular systems were constructed: $GGCX^{WT}$-FIXQ/S-MK-4 and GGCXΔArch (residues 1–606)-FIXQ/S-MK-4. The cryo-EM model of $GGCX^{WT}$ containing deprotonation of K217 and K218 was used. MK-4 was docked using AutoDock Vina[8] to generate initial structures of the protein-ligand complexes. To avoid artificial terminal interactions, truncated structures were capped with an acetyl (ACE) group at the N-terminus and an N-methylamide (NME) group at the C-terminus. The complexes were embedded into a lipid bilayer composed of POPC mixed with a minor proportion of cholesterol using the CHARMM-GUI.[9] After solvation and neutralization with 150 mM KCl, the total number of atoms in each system reached approximately 260,000.

The force fields leaprc.protein.ff19SB,[10] leaprc.lipid21,[11] leaprc.water.tip3p,[12] and the Generalized Amber Force Field (leaprc.gaff2)[13] were employed to parameterize the

protein, lipids, water molecules (including ions), and MK-4, respectively. MK-4 parameter files were generated using Antechamber,[14] and the topology and coordinate files were prepared with LEap.[14] In order to accelerate simulation speed, SHAKE and hydrogen mass repartitioning[15] were used to allow the implementation of a 4 fs timestep.

Simulation process

After energy minimization, a 4 ns pre-equilibration under isothermal-isobaric ensemble (NPT) and periodic boundary conditions at 300 K (1 atm) was conducted with heavy restraints (force k = 100 kcal/mol/Å^2). Then restraints were gradually reduced, with van der Waals cutoff of 10 Å, using the Particle Mesh Ewald (PME) method to calculate electrostatics. Minimal volume fluctuations indicated that membrane and solvent molecules were well equilibrated, and this constant volume were fixed for the subsequent production. After that, each equilibrium simulation was elongated to 400 ns. The minimization, pre-equilibration and equilibrium simulation were performed with OpenMM.[16]

To overcome the limitations of conventional MD (cMD) and to capture ligand dissociation relevant events in the GGCXΔArch–FIXQ/S–MK-4 system, accelerated MD (aMD)[17] simulations were conducted. A boost potential ΔV(r) was added to the total and dihedral potentials to enhance conformational sampling:

$$V(r)* = V(r) + \Delta V(r)$$

$$\Delta V(r) = \frac{(Ep - V(r))^2}{(\alpha P + Ep - V(r))} + \frac{(Ed - Vd(r))^2}{(\alpha D + Ed - Vd(r))}$$

where *V(r)* and *Vd(r)* are the normal total and torsion potentials, Ep and Ed are average potential and dihedral energies, and αP and αD denotes boost factors. After parameters optimization, the total number of atoms scaled by 0.20 yielded stable results. Subsequently, aMD simulations of ~160 ns in three repeats were performed using the binary file "pmemd.cuda" in Amber23.

Evaluation of Cap and H2 motions

By analyzing per-residue root-mean-square-fluctuation (RMSF) and functional domain segmentation, three regions were identified: 1) a stable TMD with small RMSF was used as a spatial reference to eliminate translational and rotational degrees of freedom from the trajectories; 2) a flexible Cap (residues 237–254) which exhibits dynamics relative to the ligand, and 3) the second α-helix in PBD-2 (H2, residues 510–524) whose motion is associated with the Cap dynamics. Cap movement was evaluated by metric the displacement of its center of mass from its initial position (denoting as d_γ). H2 dynamics were assessed via principal component analysis (PCA) of Cα atoms in each frame, with the angle ($cos\theta$) between the trajectory's PC1 vector and the initial PC1 indicating directional change. MD trajectories were conducted using MDTraj[18] for reading, superposing and root-mean-square-deviation (RMSD) calculation. The RMSF was computed based on the "rmsf" command in CPPTRAJ,[19] and PCA was implemented with Scikit-learn.[20]

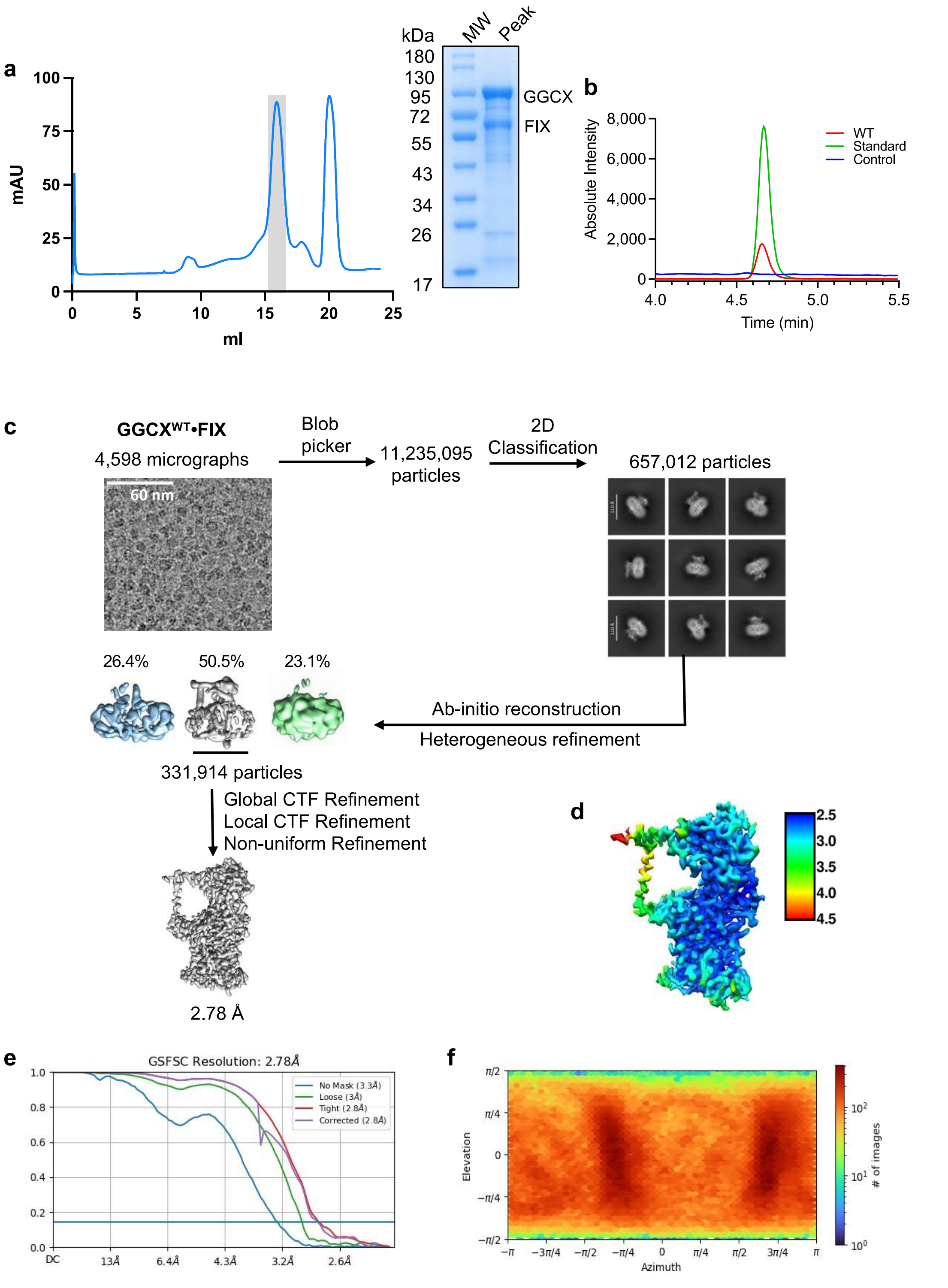

Supplementary information, Fig. S1. Cryo-EM structural analyses of GGCX^{WT}•FIX complex. a, The purification of GGCX^{WT}•FIX complex. Size-exclusion chromatography (left) and the Coomassie blue staining of SDS-PAGE (right). The gray-shaded fractions were used for cryo-EM analysis. **b,** Liquid chromatography-mass spectrometry (LC-MS/MS) analysis of vitamin K. **c,** Cryo-EM data processing

procedure accomplished in CryoSPARC. The representative results of raw micrograph and 2D classifications were shown. **d,** Local resolution map of the 2.78-Å 3D EM map. **e,** Gold-standard Fourier shell correlation (FSC) of two independent half 3D maps of $GGCX^{WT}$•FIX complex. **f,** Angular distribution of raw particles used in CryoSPARC 3D reconstruction.

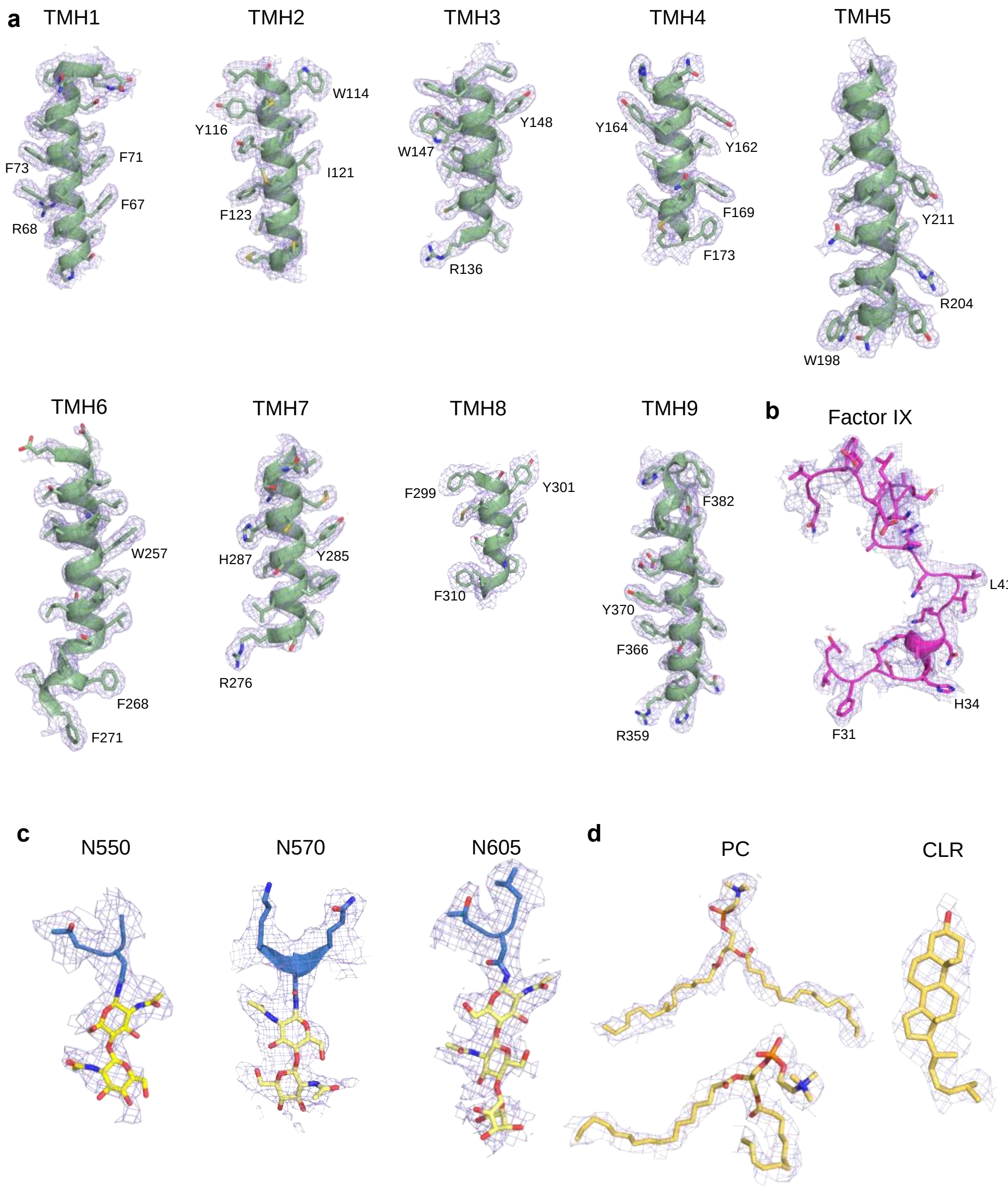

Supplementary information, Fig. S2. Representative cryo-EM densities for GGCX^{WT}•FIX complex. a–d, Density map and model in selected regions of GGCX (**a**), substrate Factor IX (FIX) (**b**), the glycosylation sites (**c**), and lipids (**d**). The sidechains of some representative residues are shown as sticks. PC, phosphatidylcholine; CLR, cholesterol.

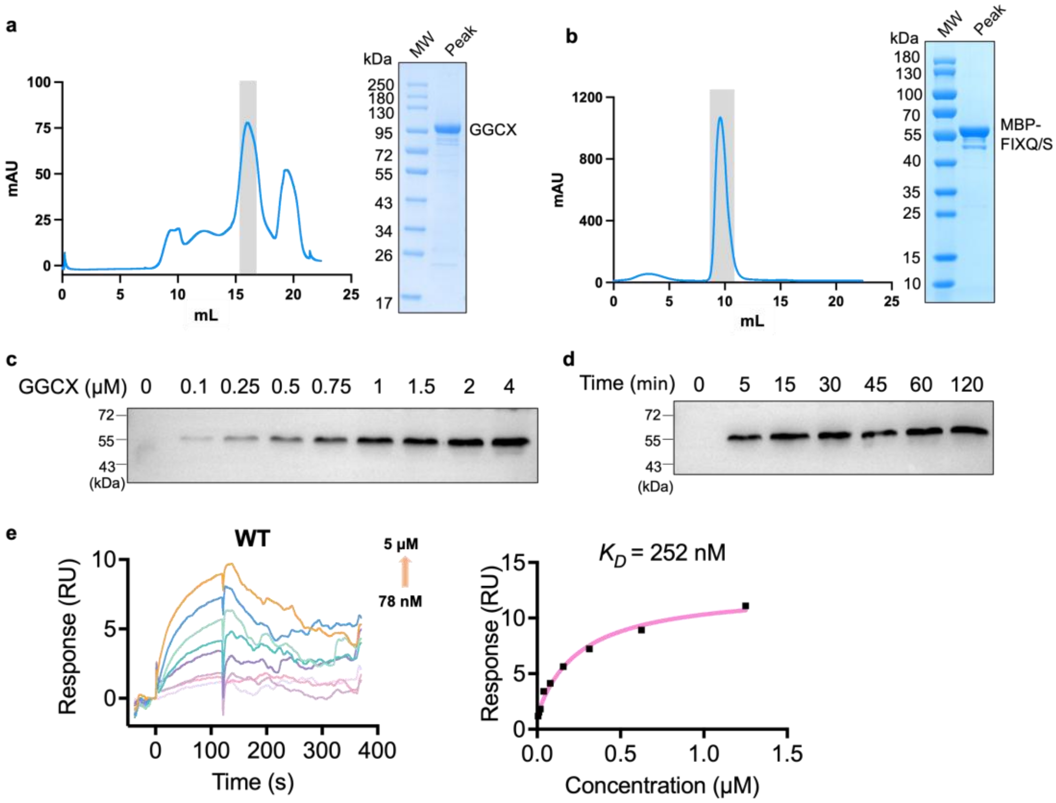

Supplementary information, Fig. S3. Purification and characterization of apo GGCX. a, Size-exclusion chromatography (left) and the Coomassie blue staining of SDS-PAGE (right) for Flag-tagged GGCX purification. The gray-shaded fractions were used for activity analysis. **b,** Purification of MBP-fused FIXQ/S (the propeptide and GLA domain of FIX, residues 29–92). **c–d,** Concentration-dependent (**c**) and time-dependent (**d**) *in vitro* reactions using MBP-FIXQ/S as the substrate. **e,** Surface plasmon resonance (SPR) analysis of GGCX binding to the propeptide and GLA domain of FIX (FIXQ/S). Raw real-time response was presented.

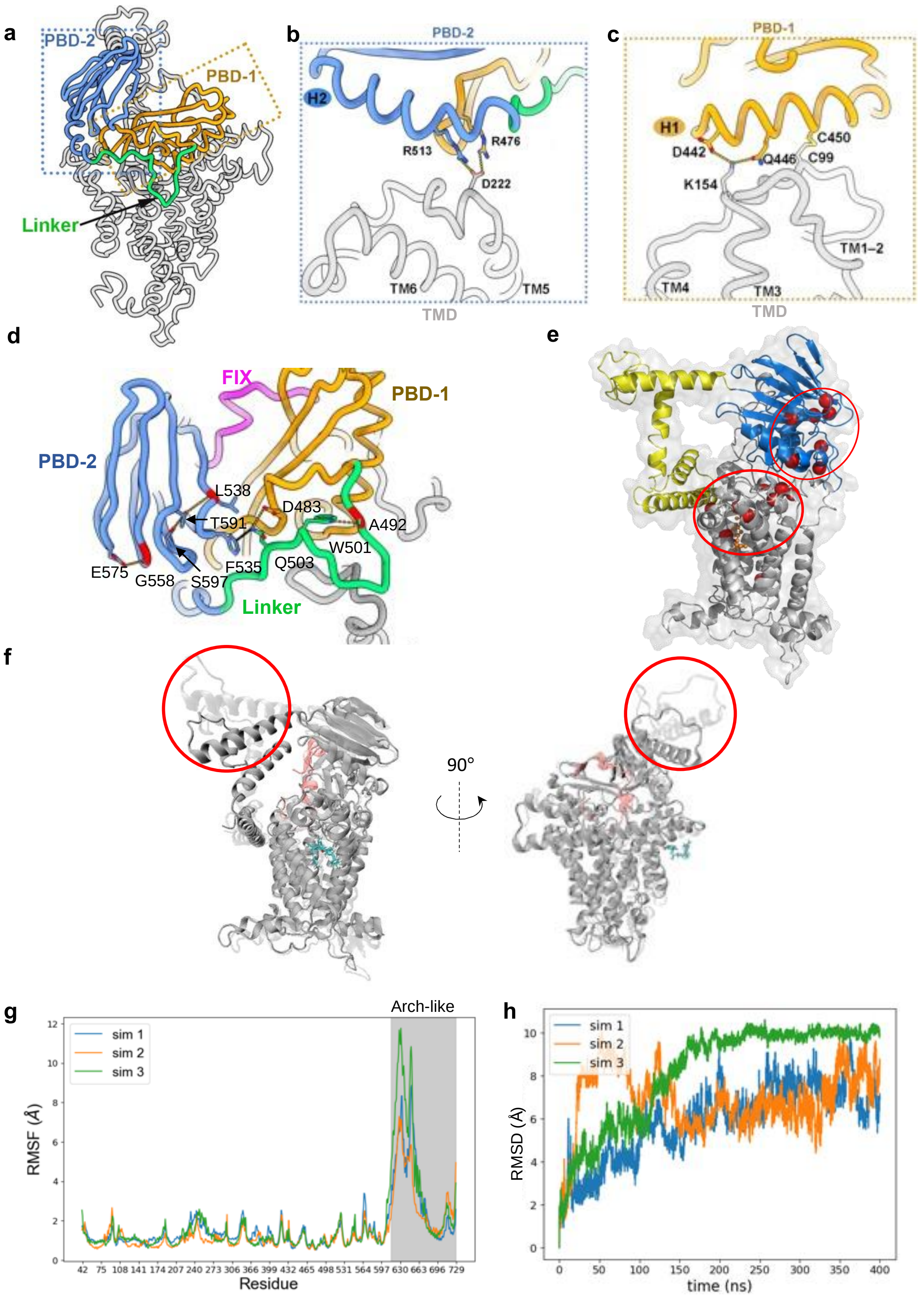

Supplementary information, Fig. S4. The composition of GGCX ER luminal domain. a, The PBD consists of two subdomains: PBD-1 (residues 405–489) and PBD-2 (residues 497–604), linked by a Linker region (residues Q490–M507). PBD-1, PBD-2, and the Linker are colored in orange, blue, and green, respectively. **b–c,** Molecular

interactions between PBD-1 (**b**) and PBD-2 (**c**) with the TMD. Residues involved in their interactions are labelled and represented as sticks. The hydrophilic interactions are indicated as yellow dashed lines. **d**, Residues locating within PBD form several molecular interactions. Hydrogen-bonds and cation-π interaction are shown as yellow and black dashed lines, respectively. **e,** Diseases-associated mutations of GGCX are mapped onto the structure and highlighted as red spheres. Two hotspots are highlighted. **f,** Structural representation of the molecular dynamics (MD) simulations using $GGCX^{WT}$-FIXQ/S-vitamin K system. Two side views of the final state are displayed, with the initial configuration of the Arch region shown in transparent mode. Conformational changes are highlighted with red circles. **g,** Per-residue root-mean-square-fluctuation (RMSF) of $GGCX^{WT}$ (excluding a few N-terminal residues and residues in loop regions) from the conventional MD (cMD) simulations. The Arch-like domain is indicated in gray. A few residues from N-terminus or loops are excluded due to their inherently flexibility. **h,** Root-mean-square-deviation (RMSD) of the Arch-like domain and the C-terminus during 400-ns MD simulations. Results from three independent simulation replicates are shown in blue, orange, and green, respectively.

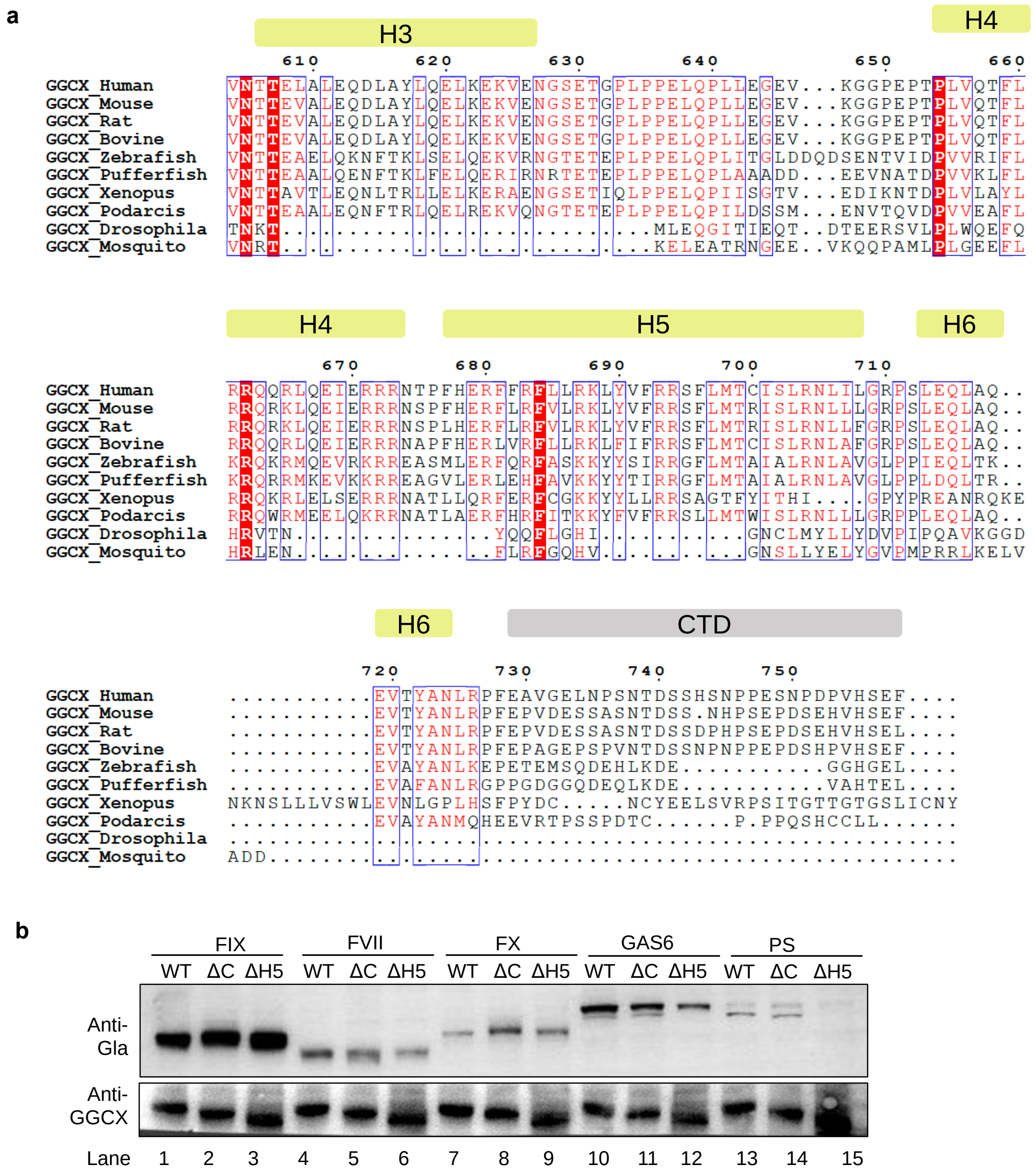

Supplementary information, Fig. S5. Sequence alignment of GGCX homologs. a, Alignment of amino acid sequences of GGCX homologs generated with Clustal Omega (Uniprot identifiers: P38435, Q9QYC7, O88496, Q07175, A0A8M1RIV5, H3CUP2, A0A6I8PZ28, A0A670IZL4, Q9NDA0, Q7Q5L8). Helix H3, H4, H5, and the unconservative C-terminus (CTD) are shown. **b,** Cell-based γ-carboxylation assays of GGCX truncation constructs, with either a C-terminal deletion (ΔC, residues 1–728) or the H5-deletion (ΔH5, residues 1–676), compared to the wild-type (WT) protein. FIX,

coagulation factor IX; FVII, coagulation factor VII; FX, coagulation factor X; GAS6, growth arrest-specific protein 6; PS, protein S.

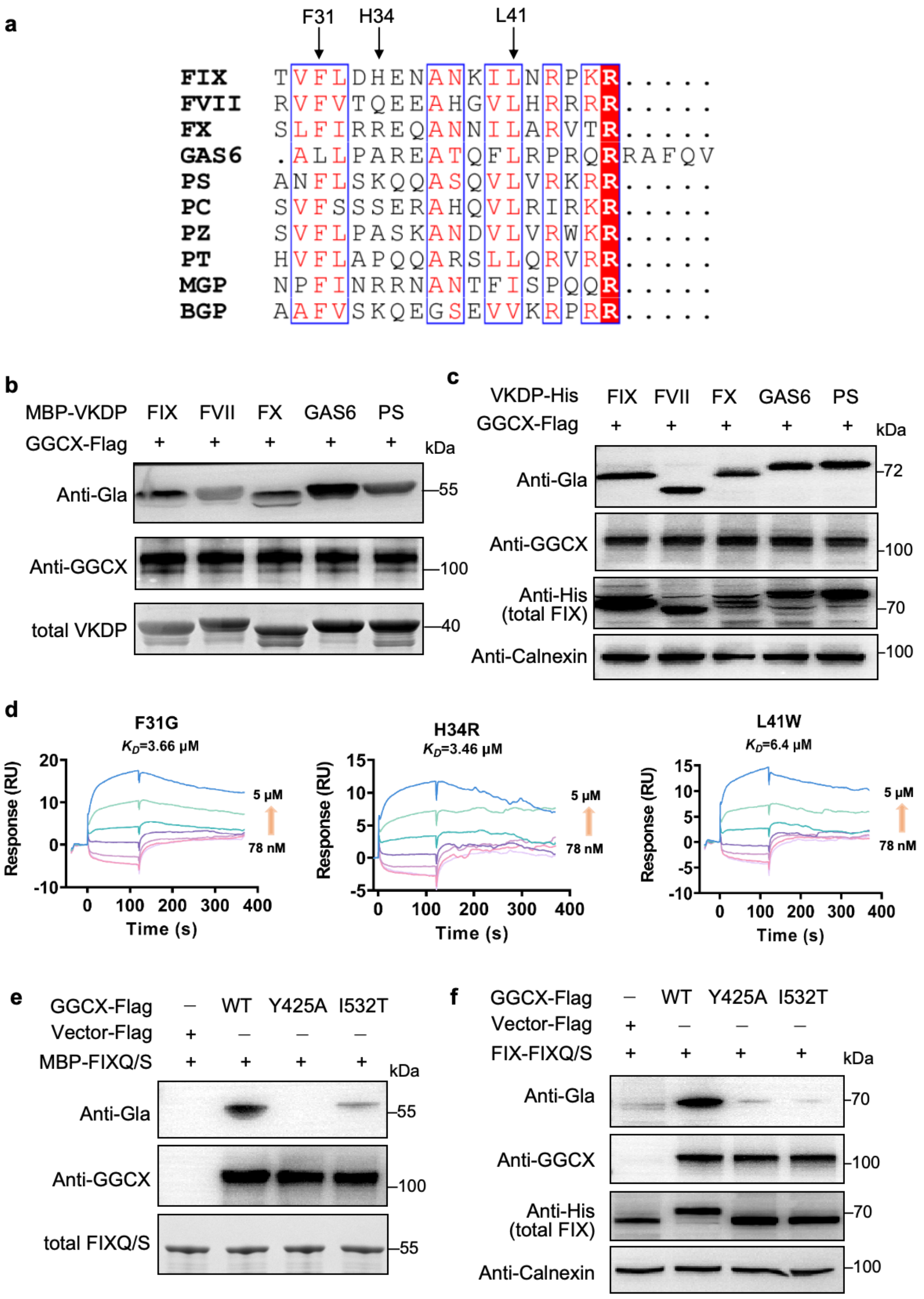

Supplementary information, Fig. S6. The binding and γ-carboxylation of various VKDPs. a, Sequence alignment for various substrates of GGCX. The conserved residues of FIX are labeled. PC, protein C; PZ, protein Z; PT, Prothrombin; MGP, matrix Gla protein; BGP, bone Gla protein. **b,** *In vitro* γ-carboxylation activity of different VKDPs. N-terminal MBP tag fused with the propeptide and Gla region of

VKDPs (MBP-VKDP) were used. **c**, Cell-based assays using full-length VKDPs fused with C-terminal His tag (VKDP-His) as substrates. **d,** Real-time response of the mutant substrates (F31G, H34R, and L41W) at different ligand concentrations against GGCX assessed by SPR. **e–f,** Carboxylation activity of MBP-FIXQ/S using GGCX in substrate-binding mutations (Y425A and I532T) measured by both *in vitro* reaction (**e**) and cell-based assay (**f**).

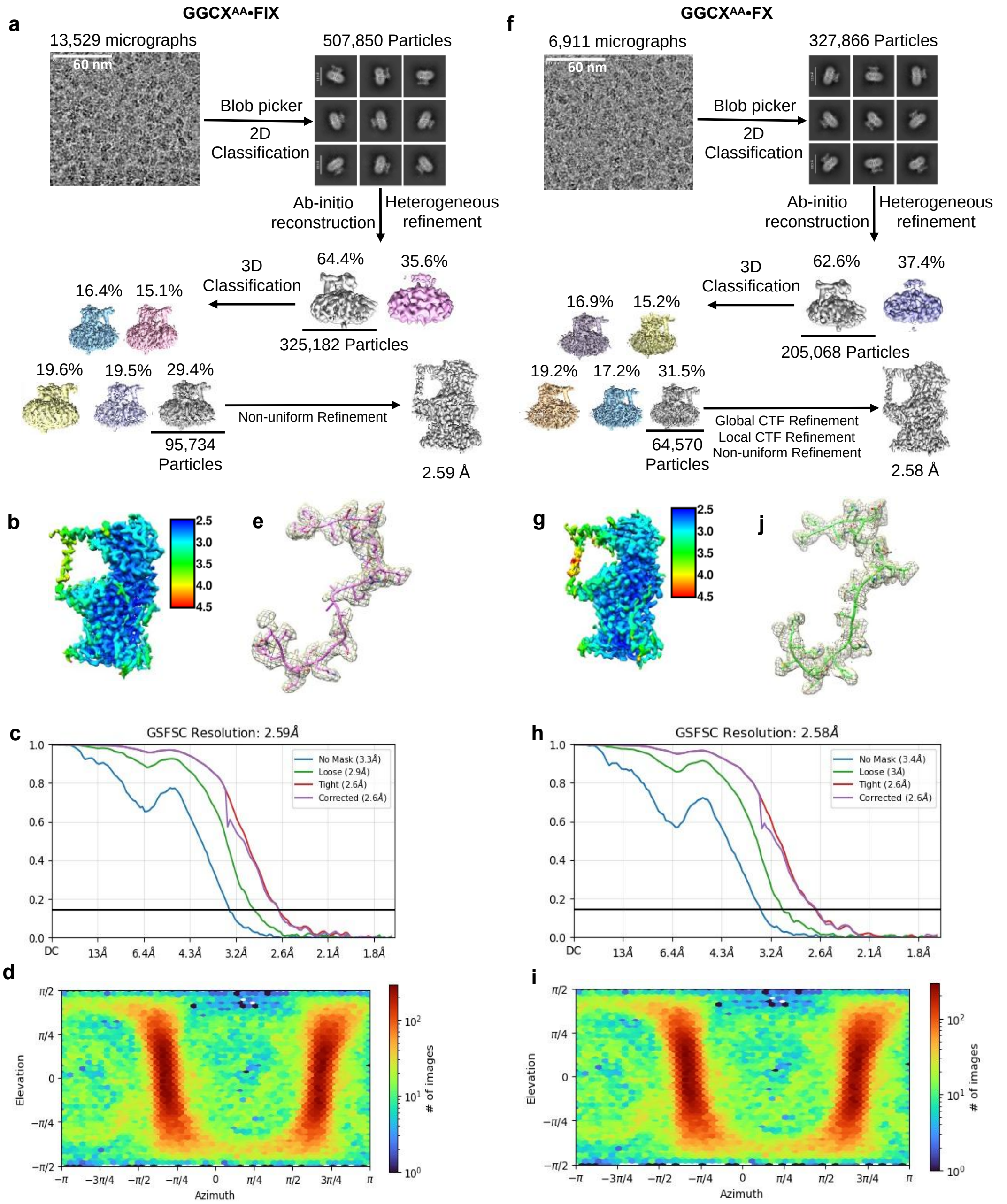

Supplementary information, Fig. S7. Cryo-EM structural determination of FIX- and FX-bound mutated GGCX. a, Cryo-EM data processing procedures for GGCX-K217A&K218A (GGCX^{AA}) in complex with FIX and Vitamin K. **b,** Local resolution map of the GGCX^{AA}•FIX complex at 2.59 Å. **c,** Gold-standard FSC of two independent half 3D maps of GGCX^{AA}•FIX complex. **d,** Angular distribution of raw particles used in CryoSPARC 3D reconstruction. **e,** EM density map and the corresponding model for the substrate FIX. **f–j,** Similar procedures were applied for structural determination of GGCX^{AA}•FX complex at 2.58 Å.

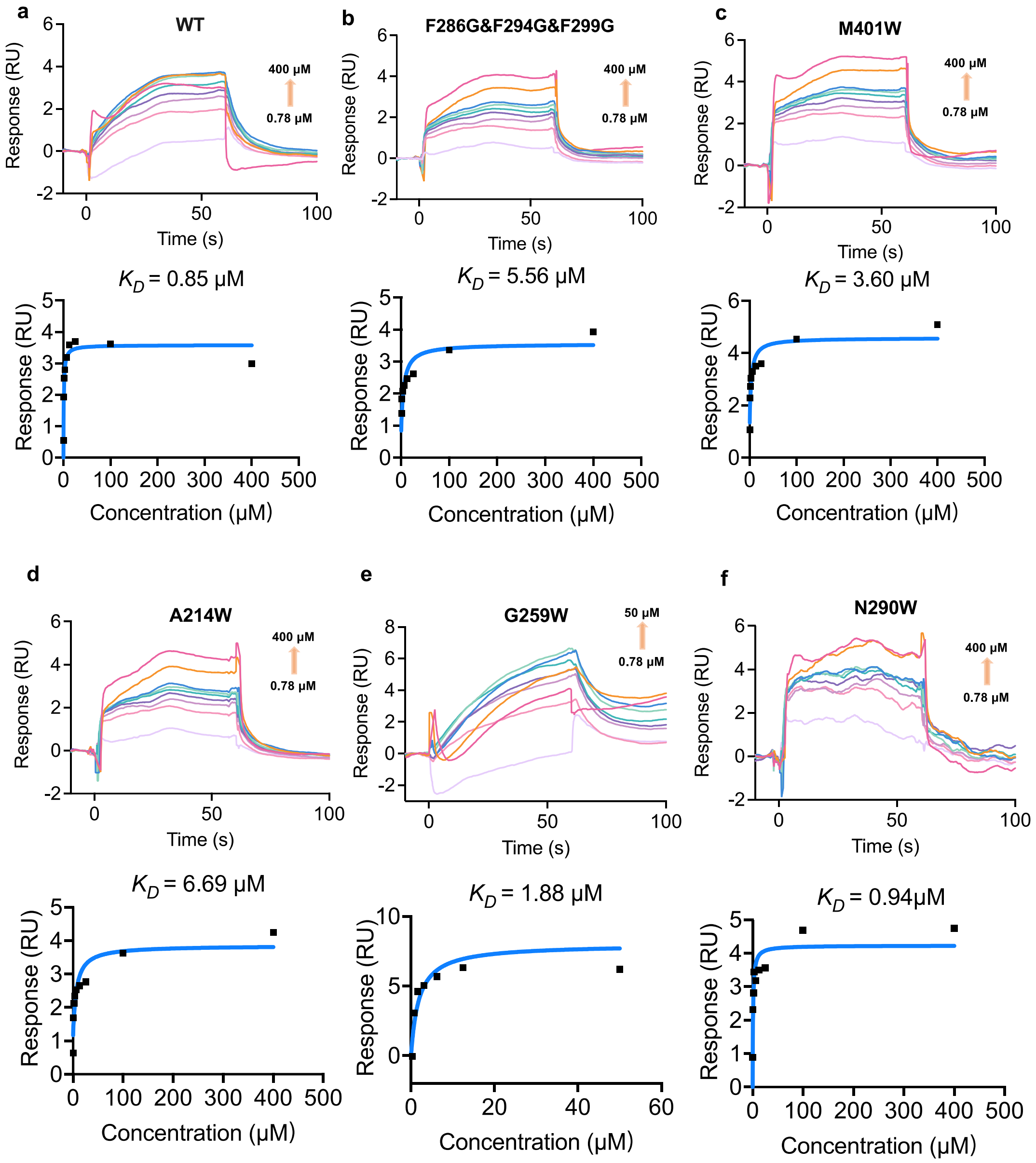

Supplementary information, Fig. S8. SPR analysis of vitamin K binding to GGCX. a–f, Real-time response of vitamin K at different ligand concentrations against WT (**a**), F286/294/299G (**b**), M401W (**c**), A214W (**d**), G259W (**e**), and N290W (**f**)**.** The used concentrations and K_D values are labelled.

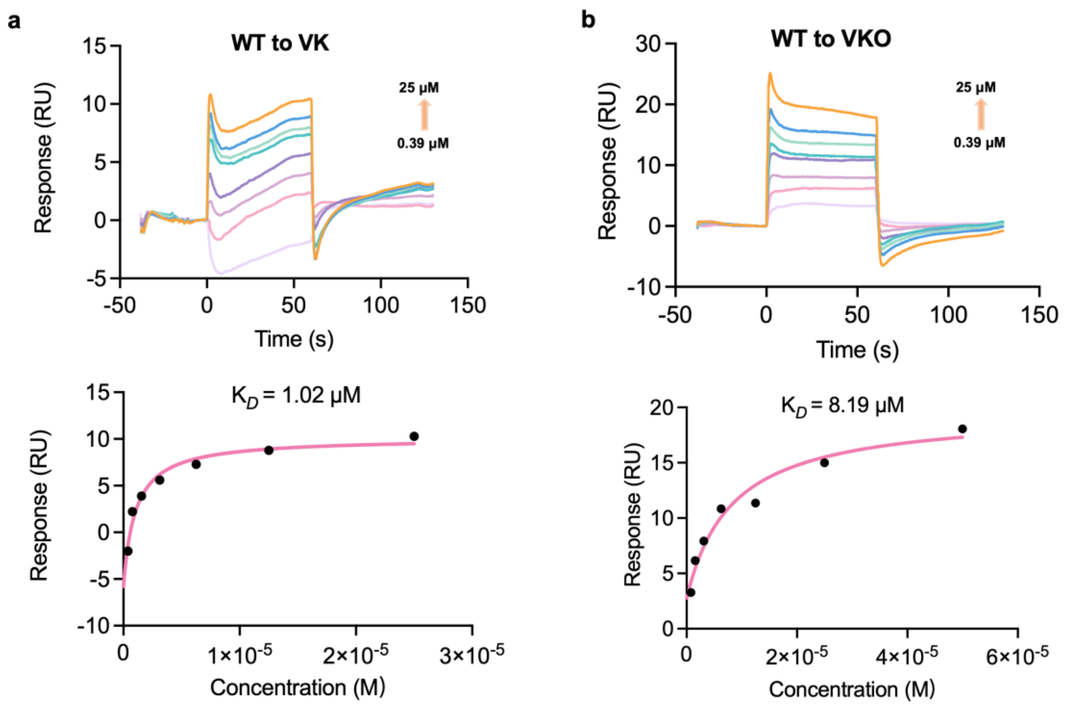

Supplementary information, Fig. S9. SPR analysis of VKO binding to GGCX compared to VK. a–b, Real-time responses and the corresponding fitted curves for binding between WT GGCX with vitamin K (**a**) and VKO (**b**).

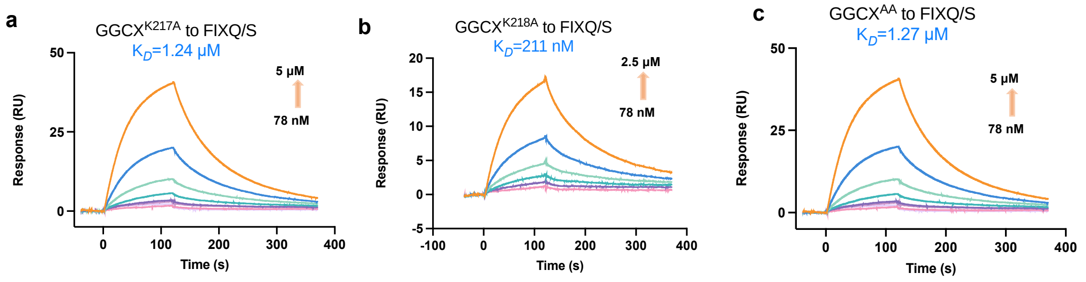

Supplementary information, Fig. S10. SPR analysis of K217A, K218A, and GGCX^{AA} to FIXQ/S. a–c, Real-time responses for binding between GGCX^{K217A} (**a**), GGCX^{K218A} (**b**) and GGCX^{AA} (**b**) and the substrate FIXQ/S.

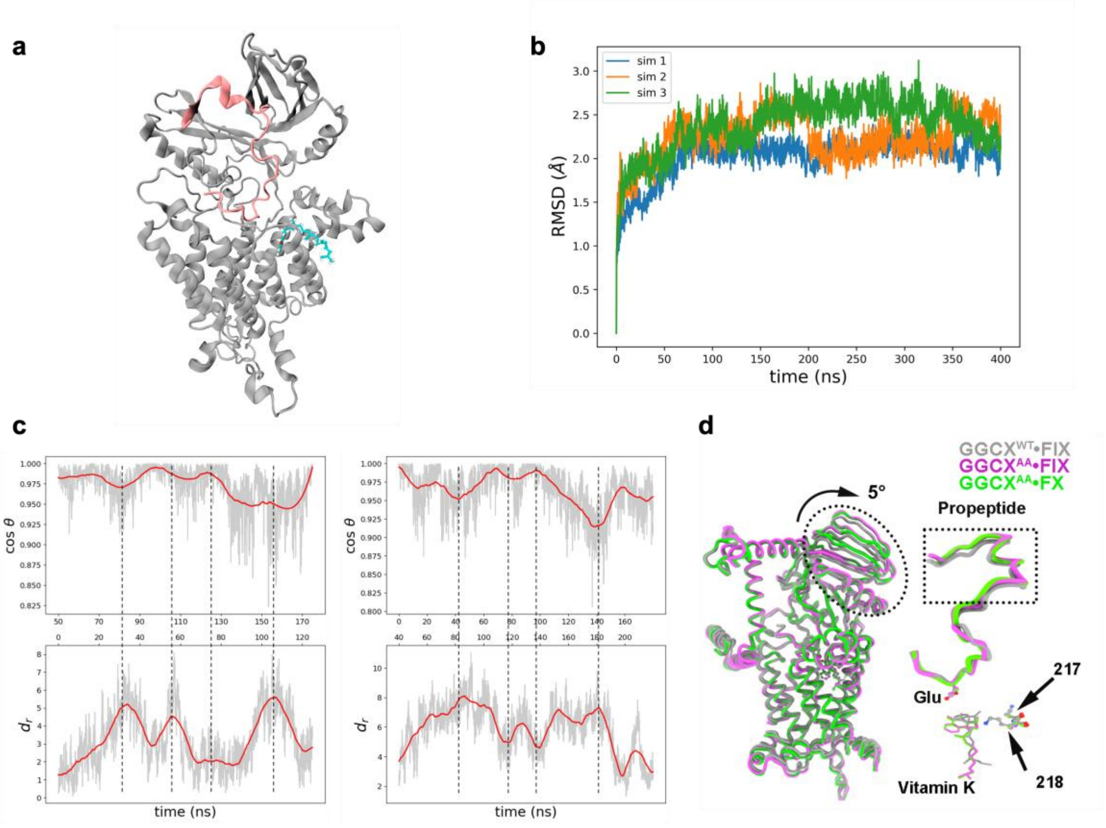

Supplementary information, Fig. S11. PBD-2 movement elucidated by MD simulation and structural comparison. a, Structural representation of GGCXΔArch (residues 1–606)-FIXQ/S-MK-4 system obtained by cMD simulations. FIXQ/S is colored in pink. **b,** Time course plot for GGCXΔArch and FIXQ/S RMSD changes during three independent repeats of 400-ns simulations. **c,** The other two independent accelerated MD (aMD) simulations similar in Fig. 1x. **d,** Superimposition of $GGCX^{AA}$•FIX (magenta) and $GGCX^{AA}$•FX (green) with the $GGCX^{WT}$•FIX (gray) structure. The rotation of PBD-2 domain is highlighted by a dashed ellipse. Overlay of the bound substrates and vitamin K is shown in right with the propeptide highlighted.

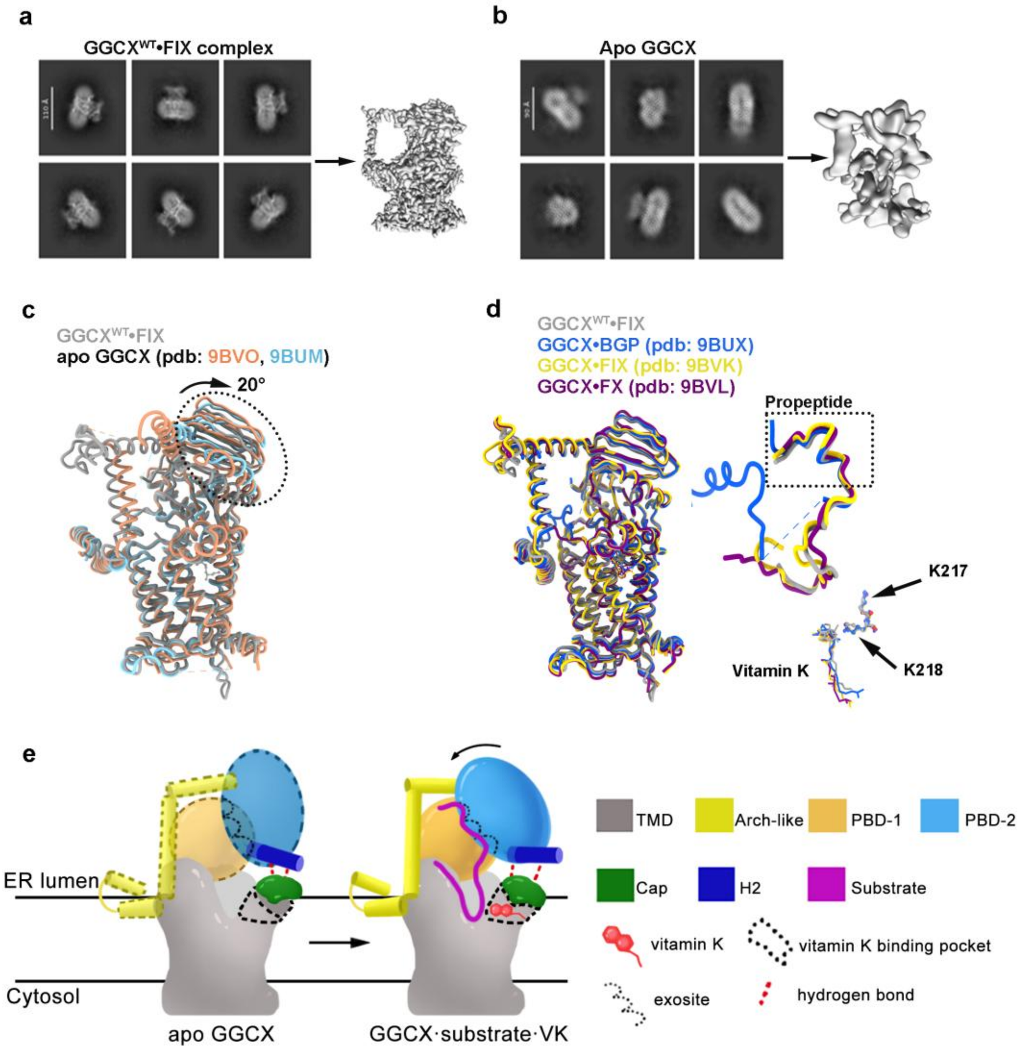

Supplementary information, Fig. S12. Working model of GGCX. a–b, Representative results of the 2D classification and 3D map of the resolved GGCX^{WT}•FIX complex (**a**) and that of apo GGCX (**b**). **c,** Superimposition of apo GGCX structures (PDB ID: 9BVO and 9BUM) onto GGCX^{WT}•FIX (gray) with the PBD-2 highlighted. **d**, Superimposition of GGCX^{WT}•FIX (gray) onto GGCX•BGP (PDB ID: 9BUX), GGCX•FIX (PDB ID: 9BVK), and GGCX•FX (PDB ID: 9BVL). The conformations of K217 and K218 remain unchanged across all complexes. **e,** The schematic model of GGCX coupling mechanism. In apo form, the luminal domain exhibits significant conformational flexibility. Substrate binding at the exosite and vitamin K binding at the binding pocket is coordinated by the synergistic H2-Cap transduction.

Supplementary information, Table S1. Cryo-EM data collection, refinement and validation.

	GGCX•FIX complex (EMD-62862; PDB 9L6Q)	$GGCX^{AA}$•FIX complex (EMD-62863; PDB 9L6R)	$GGCX^{AA}$•FX complex (EMD-62864; PDB 9L6S)
**Data collection and processing**			
Voltage (kV)	300	300	300
Microscope	FEI Titan Krios G3	Thermo Fisher Scientific Krios G4	Thermo Fisher Scientific Krios G4
Camera	Gatan K3 Summit with energy filter	Gatan BioContinuum	Gatan BioContinuum
Magnification (calibrated)	81,000 ×	105,000 ×	105,000 ×
Electron exposure ($e^-/Å^2$)	60	40	40
Exposure rate	13.10 $e^-/Å^2/s$	23.53 $e^-/Å^2/s$	23.53 $e^-/Å^2/s$
Number of frames per micrograph	32	32	32
Energy filter slit width (eV)	20	20	20
Defocus range (-μm)	0.8-1.2	0.8-1.8	0.8-1.8
Pixel size (Å)	1.07	0.85	0.85
Micrographs used	4,598	13,529	6,911
Initial particle images (no.)	11,235,095	17,399,039	7,699,519
Final particle images (no.)	331,914	95,734	64,570
Symmetry imposed	C1	C1	C1
Map resolution (Å)	2.78	2.59	2.58
FSC threshold	0.143	0.143	0.143
**Refinement**			
Resolution (Å) at 0.143 FSC of masked reconstruction	2.74	2.54	2.55

Resolution (Å) at 0.5 FSC of masked reconstruction	2.94	2.72	2.77
Model resolution range (Å)	2.83-3.07	2.57-3.21	2.55-3.16
Map sharpening B factor ($Å^2$)	-78.5	-68.3	-66.1
Model composition			
Non-hydrogen atoms	5,853	6,082	6,090
Protein residues	697	719	717
Ligands	6PL: 2	6PL: 2	6PL: 2
	LIG: 1	MKH: 1	MKH: 1
	BMA: 1	BMA: 2	BMA: 2
	CLR: 1	CLR: 1	CLR: 1
	NAG: 6	NAG: 6	NAG: 7
R.m.s. deviations			
Bond lengths (Å)	0.021	0.020	0.020
Bond angles (°)	1.034	1.028	0.853
Validation			
MolProbity score	1.89	1.62	1.77
Clashscore	13.07	7.37	8.26
Poor rotamers (%)	2.20	1.92	1.60
Ramachandran plot			
Favored (%)	98.40	98.32	97.05
Allowed (%)	1.60	1.68	2.95
Disallowed (%)	0.00	0.00	0.00
C-beta outliers (%)	0.00	0.00	0.00
CaBLAM outliers (%)	1.47	0.99	1.13

Supplementary information, Table S2. Disease-related GGCX mutations.

GGCX Domain	VKCFD-related GGCX mutations
**Transmembrane Domain (TMD)**	D31N P80L R83W/P D153G W157R G125R D153G W157R M174R R204C V255M S284P F299S S300F W315X R325Q Q374X
**Propeptide-Binding Domain**	R476C/H R485P W493C/S W501S I532T D534V G537A G558R T591K
**Arch-like Domain**	R704X
**Other Positions**	D31N L394R H404P

Supplementary information, Video S1. Conformational changes of apo GGCX measured by MD simulation. The structure of GGCX is shown in cartoon and colored gray.

Supplementary information, Video S2. Conformational changes of the GGCXΔArch-FIXQ/S measured by accelerated MD simulation. The C-terminal region and Arch domain of GGCX are truncated (GGCXΔArch). The structure of GGCXΔArch is colored gray, and the bound FIXQ/S is shown in pink.

References

1. Punjani, A. *et al.*, *Nat Methods*. **14**, 290-296 (2017)
2. Jumper, J. *et al.*, *Nature*. **596**, 583-589 (2021)
3. Pettersen, E.F. *et al.*, *Protein Sci*. **30**, 70-82 (2021)
4. Emsley, P. *et al.*, *Acta Crystallogr D Biol Crystallogr*. **66**, 486-501 (2010)
5. Liebschner, D. *et al.*, *Acta Crystallogr D Struct Biol*. **75**, 861-877 (2019)
6. Afonine, P.V. *et al.*, *Acta Crystallogr D Struct Biol*. **74**, 531-544 (2018)
7. Chen, V.B. *et al.*, *Acta Crystallogr D Biol Crystallogr*. **66**, 12-21 (2010)
8. Eberhardt, J. *et al.*, *J Chem Inf Model*. **61**, 3891-3898 (2021)
9. Wu, E.L. *et al.*, *J Comput Chem*. **35**, 1997-2004 (2014)
10. Tian, C. *et al.*, *J Chem Theory Comput*. **16**, 528-552 (2020)
11. Dickson, C.J. *et al.*, *J Chem Theory Comput*. **18**, 1726-1736 (2022)
12. Jorgensen, W. *et al.*, *J. Chem. Phys.* **79**, 926-935 (1983)
13. Wang, J. *et al.*, *J Mol Graph Model*. **25**, 247-260 (2006)
14. Case, D.A. *et al.*, *J Chem Inf Model*. **63**, 6183-6191 (2023)
15. Hopkins, C.W. *et al.*, *J Chem Theory Comput*. **11**, 1864-1874 (2015)
16. Eastman, P. *et al.*, *J Phys Chem B*. **128**, 109-116 (2024)
17. Hamelberg, D. *et al.*, *J Chem Phys*. **120**, 11919-11929 (2004)
18. McGibbon, R.T. *et al.*, *Biophys J*. **109**, 1528-1532 (2015)
19. Roe, D.R. *et al.*, *J Chem Theory Comput*. **9**, 3084-3095 (2013)
20. Kondo, Y., *Nihon Hoshasen Gijutsu Gakkai Zasshi*. **79**, 1387-1394 (2023)
